# Candidate chemosensory receptors in the antennae and maxillae of *Spodoptera frugiperda* (J. E. Smith) larvae

**DOI:** 10.3389/fphys.2022.970915

**Published:** 2022-09-15

**Authors:** Ya-Lan Sun, Peng-Shuo Jiang, Bing-Xin Dong, Cai-Hong Tian, Jun-Feng Dong

**Affiliations:** ^1^ College of Horticulture and Plant Protection, Henan University of Science and Technology, Luoyang, China; ^2^ Institute of Plant Protection, Henan Academy of Agricultural Sciences, Zhengzhou, China

**Keywords:** larval transcriptome, odorant receptor, gustatory receptor, ionotropic receptor, real-time quantitative-PCR, *Spodoptera frugiperda*

## Abstract

Although most of the damage caused by lepidopteran insects to plants is caused by the larval stage, chemosensory systems have been investigated much more frequently for lepidopteran adults than for larvae. The fall armyworm *Spodoptera frugiperda* (J. E. Smith) (Lepidoptera: Noctuidae) is a polyphagous and worldwide pest. To understand the larval chemosensory system in *S. frugiperda*, we sequenced and assembled the antennae and maxillae transcriptome of larvae in the sixth instar (larval a-m) using the Illumina platform. A total of 30 putative chemosensory receptor genes were identified, and these receptors included 11 odorant receptors (ORs), 4 gustatory receptors (GRs), and 15 ionotropic receptors/ionotropic glutamate receptors (IRs/iGluRs). Phylogeny tests with the candidate receptors and homologs from other insect species revealed some specific genes, including a fructose receptor, a pheromone receptor, IR co-receptors, CO_2_ receptors, and the OR co-receptor. Comparison of the expression of annotated genes between *S. frugiperda* adults and larvae (larval a-m) using RT-qPCR showed that most of the annotated OR and GR genes were predominantly expressed in the adult stage, but that 2 ORs and 1 GR were highly expressed in both the adult antennae and the larval a-m. Although most of the tested IR/iGluR genes were mainly expressed in adult antennae, transcripts of 3 iGluRs were significantly more abundant in the larval a-m than in the adult antennae of both sexes. Comparison of the expression levels of larval a-m expressed chemosensory receptors among the first, fourth, and sixth instars revealed that the expression of some of the genes varied significantly among different larval stages. These results increase our understanding of the chemosensory systems of *S. frugiperda* larvae and provide a basis for future functional studies aimed at the development of novel strategies to manage this pest.

## Introduction

Chemical communication is essential for various insect behaviors such as mating, feeding, foraging, and oviposition (Hildebrand and Shepherd, 1997). The chemosensory process consists of several major events, including the conversion of compounds into electrical signals at the periphery, the integration of the electrical signals into the antennae lobes (for olfaction) or the subesophageal ganglion (for gustation), and ultimately the production of behavioral signals in brain centres (Leal, 2013; Wilson, 2013; He et al., 2022). At the periphery level, the chemosensory process is mainly mediated by three families of receptor genes including odorant receptors (ORs), gustatory receptors (GRs), and ionotropic receptors (IRs) (Dahanukar et al., 2005; Hansson and Stensmyr, 2011).

Insect GRs and ORs were first identified in the model insect *Drosophila melanogaster* (Gao and Chess, 1999; Clyne, 2000). These GRs and ORs are seven transmembrane proteins (350–500 amino acids), which have a similar motif at the C-terminal (Robertson et al., 2003). Compared to the classic vertebrate G-protein coupled receptors, insect GRs and ORs have an inverted topology (Benton et al., 2006). Receptors in the OR family are classified into two types: highly conserved OR co-receptors (ORco) and a variable “tuning” receptor (ORx). Insect ORs are heterodimers composed of a unique OR and the ORco. The heterodimer acts as a ligand-gated ion channel and may also function in metabolic signal transduction pathways (Sato et al., 2008; Wicher et al., 2008; Stengl, 2010). Insect ORs have also been reported to be heterotetramers based upon the cryo-electron microscopy structure of an ORco homomer from the parasitic wasp *Apocrypta bakeri* (Butterwick et al., 2018). Insect GRs occur mostly in gustatory sensilla that contact food or other substances being tasted. GRs can detect bitter and sweet compounds as well as carbon dioxide (Scott et al., 2001). Genes in the GR family can be divided into several major clades, which include genes that encode GRs that detect carbon dioxide (Jones, et al., 2007), sugar compounds (Fujii et al., 2015), fructose (Miyamoto et al., 2012), and bitter compounds (Delventhal and Carlson, 2016).

Like studies of insect GRs and ORs, studies of insect IRs were initiated with *D*. *melanogaster*. Insect IRs have evolved from the neurotransmitter receptors for glutamate, i.e., ionotropic glutamate receptors (iGluRs) (Benton et al., 2009; Croset et al., 2010; Robertson, 2019). Insect IRs are expressed in various tissues, have diverse functions, and can be classified into two groups based on their expression profile and sequence type. The “antennal IRs” are mainly expressed in the antennae and especially in neurons of coeloconic sensilla, which have been implicated in the sensing of amino acids, acids, and amines (Silbering et al., 2011; Ai et al., 2013; Hussain et al., 2018). Most insect IRs are “divergent IRs”. Receptors in this subgroup have variable sequences and are expressed in different tissues of the insects (Croset et al., 2010). Although the majority of divergent IRs are involved in olfaction and gustation (Koh et al., 2014), some function in the sensing of sound, temperature, and humidity (Knecht et al., 2016; Frank et al., 2017). Similar to the ORs, co-expression is also observed among IRs. Insect IRs can function in complex combinations, with at least three members (IR8a, IR25a, and IR76b) acting as co-receptors with tuning IRs (Abuin et al., 2011; Chen et al., 2015; Ni et al., 2016; Ganguly et al., 2017).

Moths constitute a large group of members in the Lepidoptera. During the lifetime of moths, their adults mainly function in mating and oviposition, while the larvae mainly function in feeding and thereby cause substantial damage to crops and other plants. Control of moth pests depends on understanding the chemosensory systems of both adults and larvae. Most researchers have focused on the chemosensory mechanisms of adult moths (Montagné et al., 2012; Chang et al., 2017; Liu et al., 2020), and relatively little is known about the chemosensory mechanisms of moth larvae. However, because they have a simple chemosensory system, with antennae and maxillae being their main chemosensory organs (Dethier and Schoonhoven, 1969; Itagaki and Hildebrand, 1990), moth larvae are ideal models for research on insect chemoreception. Identifications of larval chemosensory receptor repertoires had been reported in several moth species, including (but not limited to) *Bombyx mori* (Tanaka et al., 2009), *Spodoptera littoralis* (Poivet et al., 2013), *Helicoverpa armigera* (Liu et al., 2014), *H*. *assulta* (Xu et al., 2015), and *S*. *litura* (Li et al., 2021). Among which, functions for a few specific chemosensory receptors have been characterized. Comprehensive investigation of ORs in the larval stage of the silkworm *B*. *mori* indicated that a specifically receptor, BmOR56, may mediate the attraction of the mulberry leave volatile *cis*-jasmone to the caterpillars (Tanaka et al., 2009). Tissue-specific expression using RT-PCR found nine ORs in larval *S*. *littoralis*, and behavioral investigation revealed that these ORs are related to the caterpillar attraction to nine plant volatiles (De Fouchier et al., 2018). A recent study of a putative bitter GR (BmorGR66) from *B*. *mori* shows that BmorGR66 is mainly expressed in the larval maxilla and is responsible for the larvae feeding preference (Zhang et al., 2019).

The fall armyworm *Spodoptera frugiperda* (Lepidoptera: Noctuidae) is an important pest that originated in tropical and subtropical regions in North and South America (Sparks, 1979). It invaded the United States and Canada in the middle of the 19th century (Johnson, 1987; Early et al., 2018). Because of its strong migratory ability, *S*. *frugiperda* has spread to over 40 African countries within the last 6 years (Goergen et al., 2016; Cock et al., 2017). It has also recently invaded and rapidly spread across China (Wu et al., 2019). *S. frugiperda* is highly polyphagous, and its larvae can feed on many important cultivated crops such as rice, corn, peanuts, and soybeans (Meagher and Nagoshi, 2012). Pair-wise choice tests showed that *S. frugiperda* larvae can discriminate among inbred lines of maize, and are attracted to or not attracted to larvae-infested plants depending on the plant variety (Yactayo-Chang et al., 2021). The molecular basis underlying the chemosensory system in *S. frugiperda* larvae, however, is unknown.

In this study, we sequenced the transcriptome of the antennae and maxillae of *S*. *frugiperda* larvae and identified 11 ORs, 4 GRs, and 15 IRs/iGluRs. We constructed phylogenetic trees of these receptors and homologs in other insect species in order to determine the putative functions of the candidate genes. We then used real-time quantitative-PCR (RT-qPCR) to investigate the expressional profiles of these genes in *S. frugiperda* larvae and adults. Furthermore, we analyzed the expression levels of varoius chemosensory receptor genes at different larval development stages using RT-qPCR. The results provide a foundation for further studies of the functions of the chemoreceptors in *S. frugiperda* larvae and for the development of novel methods to control this pest.

## Materials and methods

### Insect rearing

*Spodoptera frugiperda* larvae were collected from a corn field in Shidian County, Baoshan, Yunnan Province, China. They were then reared in the laboratory at Henan University of Science and Technology, Luoyang, China. The insects were reared in a climatic cabinet at 26 ± 1°C with a relative humidity of 70% ± 5% and a photoperiod of 16 h: 8 h (L: D). The larvae were fed with an artificial diet that mainly contained wheat germ, corn leaf powder, and yeast powder. Male and female pupae were placed in separate cages for emergence. Adults were supplied with a 15% honey solution (v/v). Seven male moths and eight female moths were kept in a net cage for mating and oviposition.

### Tissue collection and total RNA extraction

For transcriptome sequencing and RT-qPCR, we dissected the head of 200 *S. frugiperda* larvae in the first instar (1 day after hatching), the antennae and maxillae of 400 larvae in the fourth instar (second day in the fourth instar), the antennae and maxillae of 400 larvae in the sixth instar (second day in the sixth instar), the antennae of 80 adult males (3 days after eclosion), and the antennae of 80 adult females (3 days after eclosion). The five kinds of samples were immediately immersed in liquid nitrogen and were subjected to total RNA extraction with the RNeasy Plus Mini Kit (Qiagen, Venlo, Netherlands) according to the manufacturer’s instructions. The purity and concentration of the total RNA were determined based on the OD_230_, OD_260_, and OD_280_ values as measured with a spectrophotometer (Nano Drop 2000; Nano-Drop Products, Wilmington, DE, United States). The extracted RNA was used for transcriptome sequencing and RT-qPCR as described in the following sections. The following sections also indicate the numbers of replications used for each determination.

### Transcriptome sequencing and assembly

According to the age-stage analysis of *S*. *frugiperda*, there are six instars during the larval period, the fifth and sixth instars are assigned to gluttonous stage, and duration of the sixth instar is the longest among all the larval development stages (Dai et al., 2020; Wang et al., 2020; Xie et al., 2021). Similar development stages were observed on artificial diet-fed *S*. *frugiperda* in our study. Therefore, Illumina sequencing of the total RNA from the larval antennae and maxillae of *S*. *frugiperda* on the second day in the sixth instar (the tissue is hereafter specifically referred to as the “larval a-m”) was performed at Sangon Biotech (Shanghai, China) using methods that were similar to those previously described (Sun et al., 2020). A 10-µg quantity (concentration ≥50 ng/μl) of total RNA was used for the synthesis of cDNA. Three biological replications were conducted. The cDNA libraries were then prepared, and paired-end reads were obtained from the cDNA libraries using the Illumina HiSeq 2000 platform. Sequence assembly was carried out with the transcriptome *de novo* assembly program (https://github.com/trinityrnaseq/trinityrnaseq/). TGICL (TGI Clustering) and Cap3 tools were then used to cluster the Trinity outputs and to thereby produce the unigenes.

### Identification and annotation of chemosensory receptors

Annotation of unigenes was performed as previously described (Sun et al., 2020). To retrieve proteins with the highest sequence similarity (E-values < 1e-5), we conducted BLAST searches against the sequences in the non-redundant (Nr) database in the NCBI, and in the COG (Clusters of Orthologous Groups of proteins), Swiss-Prot (http://www.ebi.ac.uk/uniprot), KEGG (Kyoto encyclopedia of genes and genomes), and GO (gene ontology) databases (Ashburner et al., 2000; Kanehisa, 2004; Xie et al., 2011).

Alignments comparing transcripts encoding putative larval ORs, GRs, and IRs/iGluRs were manually performed using the BLASTx tool in NCBI. The open reading frames (ORFs) of candidate chemosensory receptor genes were predicted using the Translate program (http://web.expasy.org/translate/). The full-length transcripts were determined by considering the BLASTx results as well as the start and stop codons.

The expression levels of annotated genes were estimated using the transcripts per kilobase of exon per million mapped (TPM) method (http://deweylab.github.io/RSEM/). The average TPM value of three biological replications of the sample was calculated.

### Phylogenetic analysis

The OR phylogenetic tree was built based on amino acid sequences from the datasets of insect species including *S. frugiperda* (this study), *H. armigera*, and *B. mori*. The GR phylogenetic tree was built based on amino acid sequences from the datasets of *S. frugiperda* (this study), *B. mori*, *H. armigera*, and *Danaus plexippus*. The IR/iGluR phylogenetic tree was built based on the amino acid sequences from the datasets of *S. frugiperda* (this study), *Dendrolimus punctatus*, *H. armigera*, and *D. melanogaster*. Sequences were aligned and neighbor-joining trees were constructed using MEGA11 as previously described (Sun et al., 2021). The trees were subsequently visualized and edited with Figtree v1.4.2 (http://tree.bio.ed.ac.uk/software/figtree/). The amino acid sequences of ORs, GRs, and IRs/iGluRs used in phylogenetic analyses are listed in Supplementary Table S1.

### RT-qPCR

RT-qPCR was firstly conducted to compare the relative expression levels of annotated *S. frugiperda* chemosensory receptor genes in the larval a-m, adult male antennae, and adult female antennae. Expression level analyses of the larval a-m expressed genes were then conducted in the first instar larval head (too small to separate the antennae and maxillae), the fourth instar larval antennae and maxillae, and the sixth instar larval antennae and maxillae. The *S. frugiperda β*-*actin* gene was used as the internal reference for normalizing the expression levels of the target genes. Three biological reactions were conducted for each of the three kinds of tissue samples, and each reaction was performed three times as technical replicates. Melting curves were then observed, and five PCR products were randomly selected for sequencing to avoid non-specific amplifications. Relative expression levels of all tested genes were calculated using the 2^−ΔCt^ method (Schmittgen and Livak, 2008).

The data were analyzed and figures were made with GraphPad Prism 6 (GraphPad Software Inc., San Diego, CA). The primers used in the RT-qPCR assays were designed using Primer Premier 5.0 software (PREMIER Biosoft International) and are listed in Supplementary Table S2.

## Results

### Larval transcriptome and annotation

In this study, we generated a transcriptome of the *S*. *frugiperda* larval a-m using Illumina sequencing. An average of 56.52 million clean reads were collected, and the average Q30 base ratio and GC base ratio were 90.32% and 46.98%, respectively (Supplementary Table S3). A total of 96,197 unigenes were finally assembled, with a mean length of 633 bp and an N50 length of 972 bp. Among the 96,197 unigenes, 14,171 (14.73%) were >1000 bp (Supplementary Table S4). Among the 96,197 unigenes, 44.23% (42,548) had matches (E-value < 1e-5) in the NR database. The highest homology was with *S*. *frugiperda* (33,165 unigenes, 77.95%), followed by *S. litura* (2,185 unigenes, 5.14%), *S*. *exigua* (1063 unigenes, 2.50%), and *H*. *armigera* (845 unigenes, 1.98%) (Supplementary Figure S1).

Among the 96,197 unigenes in *S*. *frugiperda* larval a-m, 16,227 (16.87%) corresponded to at least one GO category. Among the 16,227 unigenes, 7,902 were assigned to the “molecular function” (48.7%), 11,829 to the “biological process” (72.9%), and 9,719 to the “cellular component” (59.9%). The three most represented terms were “binding”, “cellular process”, and “cell” (Supplementary Figure S2).

### Candidate ORs

A total of 11 candidate OR genes were identified in the *S*. *frugiperda* larval a-m transcriptome (Table 1). For convenience, we numbered the identified chemosensory receptors in this study according to the numbers previously used for *S*. *frugiperda* sequences (whenever possible) or for best matched homologs in other moth species. The putative chemosensory receptors displayed 99%–100% amino acid sequence identities to the sequences of the chemosensory receptors reported in the genome analysis of *S*. *frugiperda* (Gouin et al., 2017). All of the identified *SfruORs* have complete ORFs based on the presence of start and stop codons, and on the results of BLASTx alignment to other lepidopteran ORs. Their nucleotide/amino acid sequences and GenBank accession numbers are listed in Supplementary Table S5.

**TABLE 1:** Unigenes of candidate chemosensory receptors in larval antennae and maxilla of *S. frugiperda*.

Name	ORF (aa)	TPM	BLASTx best hit (GenBank accession/name/species)	Full length	Identity (%)	E-value
ORs						
SfruORco	473	0.72	AAW52583.1| odorant receptor coreceptor [*Spodoptera exigua*]	Yes	99	0.0
SfruOR10	369	0.39	AZB49424.1| olfactory receptor 10 [*Heortia vitessoides*]	Yes	80	0.0
SfruOR11	435	0.37	AGI96749.1| olfactory receptor 11 [*Spodoptera litura*]	Yes	96	0.0
SfruOR12	453	0.11	AGG08878.1| putative olfactory receptor 12 [*Spodoptera litura*]	Yes	95	0.0
SfruOR15	409	0.02	XP_035429477.1|odorant receptor 4-like [*Spodoptera frugiperda*]	Yes	100	0.0
SfruOR20	381	0.01	AVF19632.1| putative odorant receptor 20 [*Peridroma saucia*]	Yes	78	0.0
SfruOR25	401	0.05	XP_035431838. |1odorant receptor 4-like [*Spodoptera frugiperda*]	Yes	100	0.0
SfruOR42	442	0.31	AIG51888.1| odorant receptor OR42 [*Helicoverpa armigera*]	Yes	86	0.0
SfruOR46	391	0.38	XP_022817447.1| odorant receptor 46a-like [*Spodoptera litura*]	Yes	97	0.0
SfruOR51	400	0.69	AGG08876.1| putative olfactory receptor 51 [*Spodoptera litura*]	Yes	95	0.0
SfruOR85	398	0.08	XP_022826861.1| odorant receptor 85c-like [*Spodoptera litura*]	Yes	95	0.0
GRs						
SfruGR1	464	0.22	XP_022828173.1| gustatory and odorant receptor 22 [*Spodoptera litura*]	Yes	99	0.0
SfruGR2	433	0.18	XP_035439638.1| gustatory and odorant receptor 22-like [*Spodoptera frugiperda*]	Yes	99	0.0
SfruGR3	475	0.34	XP_022815658.1| gustatory and odorant receptor 24 [*Spodoptera litura*]	Yes	100	0.0
SfruGR9	488	0.04	XP_035448630.1| gustatory receptor for sugar taste 43a-like [*Spodoptera frugiperda*]	Yes	100	0.0
IRs/iGluRs						
SfruIR21a	852	0.10	XP_035448875.1| ionotropic receptor 21a-like [*Spodoptera frugiperda*]	Yes	100	0.0
SfruIR25a	918	0.91	XP_035450399.1| ionotropic receptor 25a-like [*Spodoptera frugiperda*]	Yes	100	0.0
SfruIR41a	537	0.13	ADR64681.1| ionotropic receptor IR41a [*Spodoptera littoralis*]	Yes	88	0.0
SfruIR75a	603	0.77	XP_035434833.1| ionotropic receptor 75a-like [*Spodoptera frugiperda*]	Yes	80	0.0
SfruIR76b	552	0.19	ADR64687.1| ionotropic receptor IR76b [*Spodoptera littoralis*]	Yes	97	0.0
SfruIR93a	548	0.15	XP_022828312.1| ionotropic receptor 93a [*Spodoptera litura*]	No	98	0.0
SfruiGluR2	485	0.01	AIG51925.1| ionotropic glutamate receptor [*Helicoverpa armigera*]	No	97	0.0
SfruiGluR4a	853	0.04	QHB15337.1| ionotropic receptor 4 [*Peridroma saucia*]	Yes	99	0.0
SfruiGluR4b	905	0.96	XP_022827828.1| glutamate receptor ionotropic [*Spodoptera litura*]	Yes	100	0.0
SfruiGluR6	903	0.90	XP_022828316.1| glutamate receptor ionotropic [*Spodoptera litura*]	Yes	98	0.0
SfruiGluR7	419	2.95	XP_022835341.1| glutamate receptor ionotropic [*Spodoptera litura*]	No	100	0.0
SfruiGluR8	414	0.25	XP_035445289.1| glutamate receptor ionotropic [*Spodoptera frugiperda*]	No	100	0.0
SfruiGluR9	906	1.13	XP_026746544.1| glutamate receptor ionotropic [*Trichoplusia ni*]	Yes	99	0.0
SfruiGluR10	916	0.05	XP_035450107.1| glutamate receptor ionotropic [*Spodoptera frugiperda*]	Yes	100	0.0
SfruiGluR12	649	3.05	XP_022835061.1| glutamate receptor ionotropic [*Spodoptera litura*]	No	94	0.0

**FIGURE 1:**
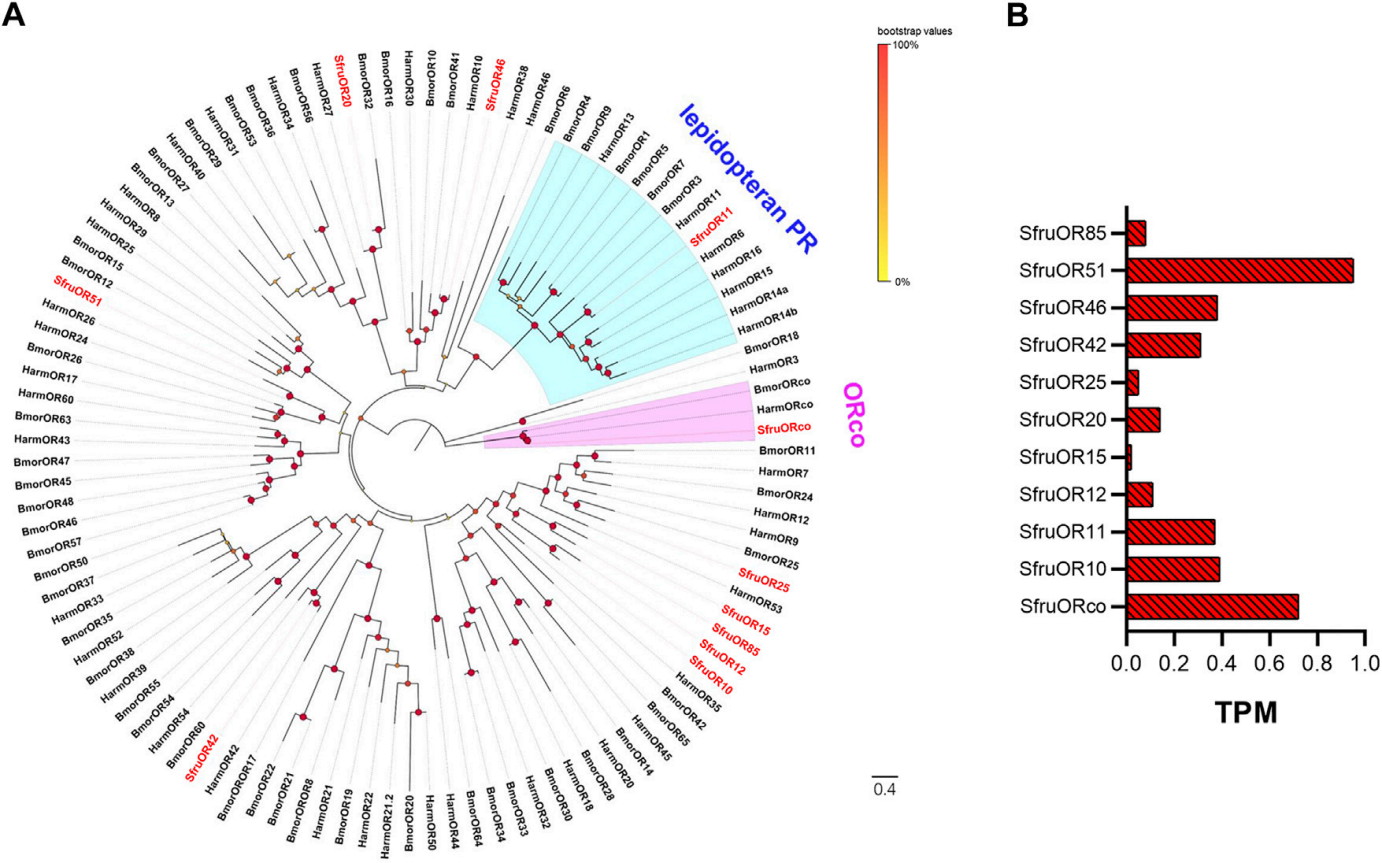
Candidate ORs of *S. frugiperda* larvae. **(A)** Phylogenetic relationships of ORs from *S. frugiperda* (Sfru, this study), *B. mori* (Bmor), and *H. armigera* (Harm). The neighbor-joining tree was constructed using MEGA11 (1000 bootstrap replicates). The tree was rooted by the ORco orthologs. The ORco clade is highlighted in pink; the lepidopteran pheromone receptor (PR) branches are highlighted in blue. **(B)** TPM values of candidate ORs in antennae and maxillae of *S. frugiperda* larvae.

A phylogenetic tree conducted with the OR dataset of *S*. *frugiperda* (this study), *H*. *armigera*, and *B*. *mori* revealed that we identified the ORco gene in the *S*. *frugiperda* larval a-m. We also identified a member (*SfruOR11*) of the lepidopteran PR subfamily, whose homologs in *H*. *armigera* and *B*. *mori* are involved in sex pheromone perception (Sakurai et al., 2004; Nakagawa et al., 2005; Liu et al., 2013; Jiang et al., 2014). Five ORs (SfruOR20, SfruOR25, SfruOR42, SfruOR46, and SfruOR51) appeared in different branches of the phylogenetic tree. The other four ORs (SfruOR10, SfruOR12, SfruOR15, and SlitIR85) clustered in an *S*. *frugiperda*-specific OR branch (Figure 1A).

The expression levels of the 11 candidate *SfruOR* genes in the larval a-m were evaluated using the TPM method. The results indicated that *SfruORco* was the most abundantly expressed OR (TPM = 0.72). *SfruOR51* had the second highest expression level, with a TPM value of 0.69. The candidate PR, *SfruOR11*, had a modest TPM value of 0.37, which was only slightly lower than the value of the third most highly expressed OR, *SfruOR46* (TPM = 0.38) (Table 1; Figure 1B).

**FIGURE 2:**
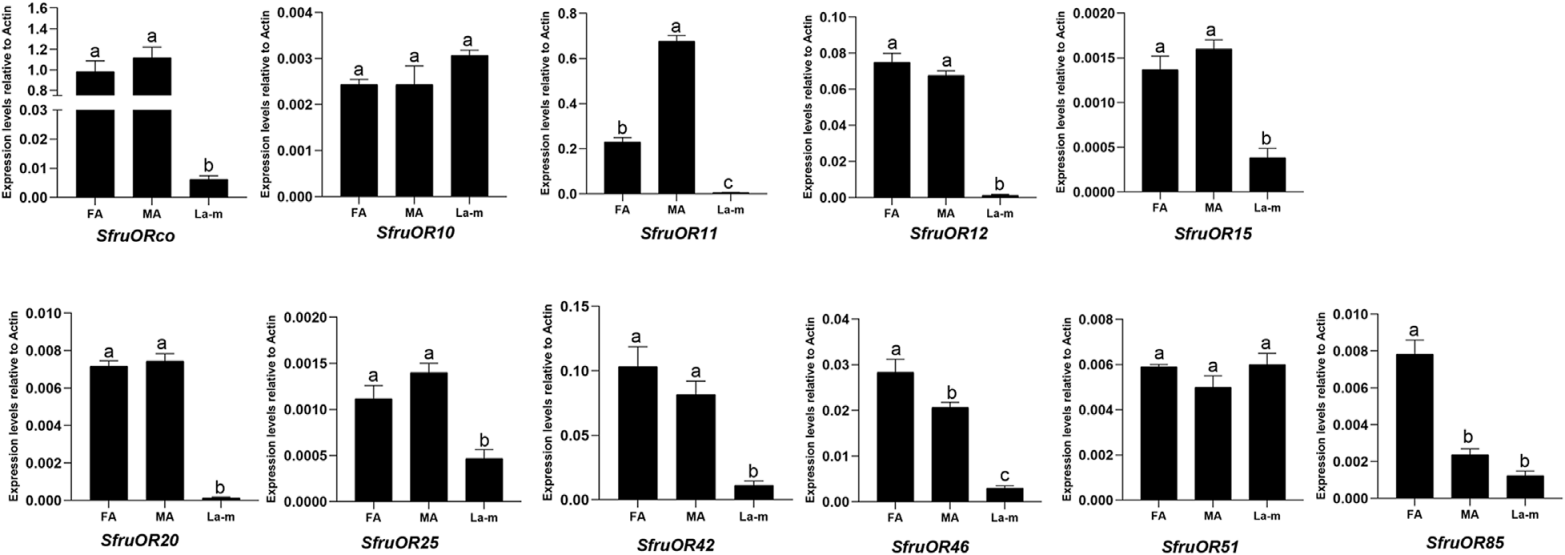
Expression profiles of candidate *SfruORs* in *S. frugiperda* larvae and adults. RT-qPCR analysis of candidate *OR* genes in male antennae (MA), female antennae (FA), and larval antennae and maxillae (L a-m). Values are means ± SE. Different letters indicate significant differences (Tukey’s multiple comparison test. after a one-way ANOVA, *p* < 0.05, *n* = 3).

Expression patterns of the 11 candidate *SfruORs* in *S*. *frugiperda* adult male antennae, adult female antennae, and the larval a-m were examined by RT-qPCR. The results showed that the expression levels of most of the tested *SfruORs* were higher in the adult antennae than in larval a-m, that expression of *SfruOR46* and *SfruOR85* was greater in female than in male antennae, and that the expression of *SfruOR11* was greater in male than in female antennae. Three transcripts, *SfruOR11*/*12*/*20*, appeared to be adult-specific, i.e., their expression was barely detected in larval a-m. The expression levels of two *Sfru*ORs (*SfruOR10* and *SfruOR51*) were similar to or tended to be slightly higher in the larval a-m than in the adult antennae of both sexes (Figure 2).

### Candidate GRs

**FIGURE 3:**
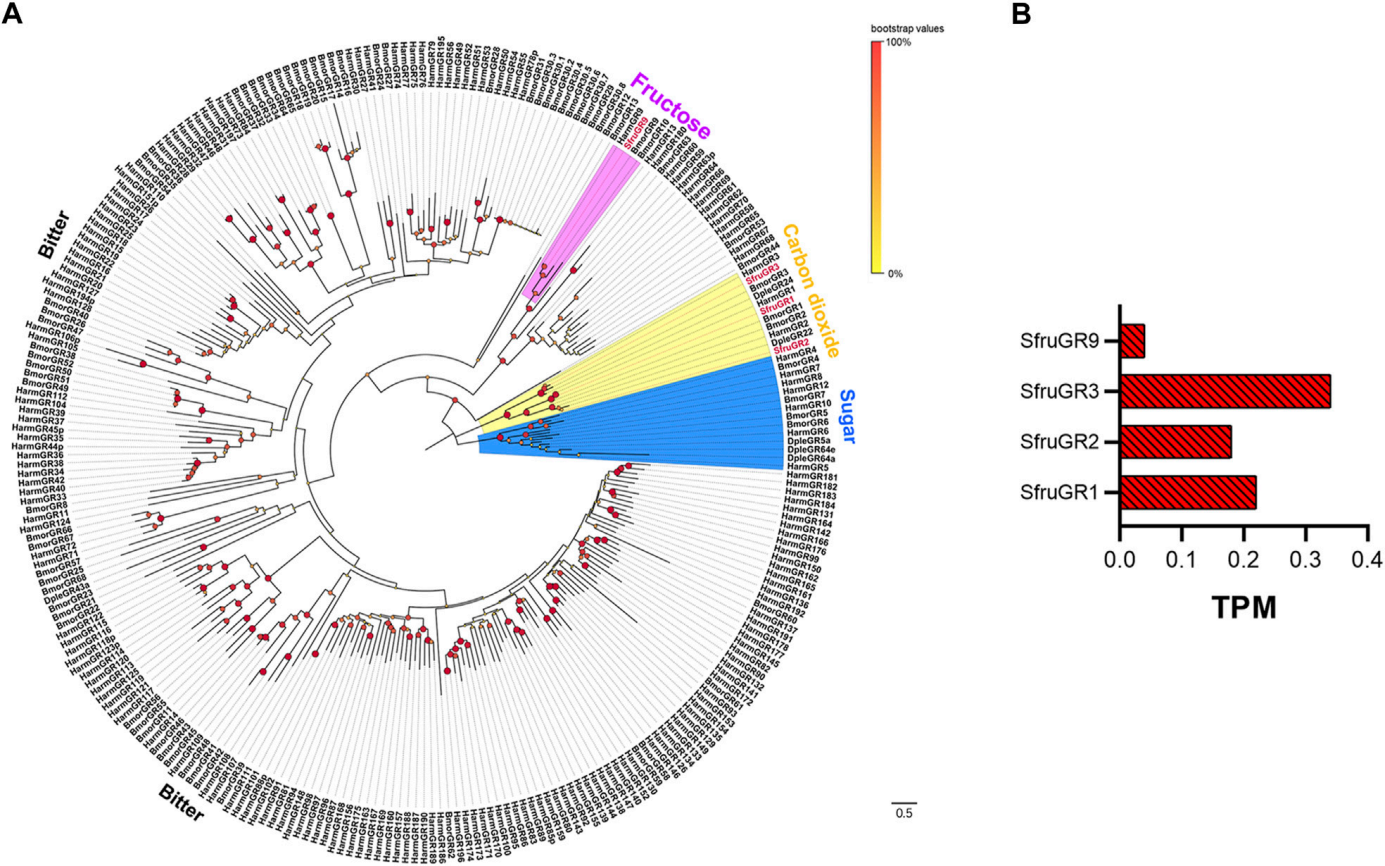
Candidate GRs of *S*. *frugiperda* larvae. **(A)** Phylogenetic relationships of GRs from *S. frugiperda* (Sfru, this study), *B. mori* (Bmor), *H. armigera* (Harm), and *D. plexippus* (Dple). The neighbor-joining tree was constructed using MEGA11 (1000 bootstrap replicates). The tree was rooted with the conservative fructose receptors. The “carbon dioxide receptor” branches are highlighted in yellow; the “fructose receptors” are highlighted in pink; the “sugar-taste receptors” are highlighted in blue; and the “bitted-taste receptor” branches are not highlighted. **(B)** TPM values of candidate GRs in antennae and maxillae of *S. frugiperda* larvae.

We identified a total of 4 candidate unigenes encoding SfruGRs in the *S*. *frugiperda* larval a-m transcriptome, and all four had full-length ORFs (Table 1, Supplementary Table S5). Phylogenetic analysis based on amino acid sequences of GRs from *S*. *frugiperda* (this study), *H*. *armigera*, *B*. *mori*, and *D. plexippus* showed that three SfruGRs (SfruGR1/2/3) were grouped with HarmGR1/2/3 and BmorGR1/2/3, which are candidate “CO_2_ receptors”. Another GR, SfruGR9, was clustered in the “fructose receptors” branch (Table1; Figure 3A), whose orthologs in *H*. *armigera* (amino acid identity 96%) and *B*. *mori* (amino acid identity 64%) were previously demonstrated to be involved in the perception of fructose (Sato et al., 2011; Xu et al., 2012; Jiang et al., 2015).

According to TPM values, the most transcribed gene among the four putative *SfruGRs* was *SfruGR3* (TPM = 0.34), which was followed by *SfruGR1* (TPM = 0.22) and *SfruGR2* (TPM = 0.18). The least transcribed of the putative *SfruGRs* was *SfruGR9* (TPM = 0.04) (Table1; Figure 3B).

**FIGURE 4:**
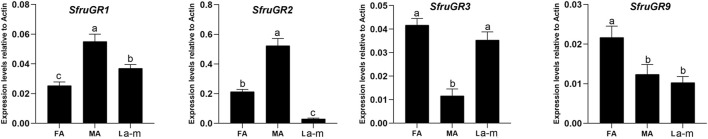
Expression profiles of candidate *SfruGRs* in *S. frugiperda* larvae and adults. RT-qPCR analysis of candidate *GR* genes in male antennae (MA), female antennae (FA), and larval antennae and maxillae (L a-m). Values are means ± SE. Different. letters indicate significant differences (Tukey’s multiple comparison test after a one-way ANOVA, *p* < 0.05, *n* = 3).

The four candidates *SfruGRs* were found to be expressed in all three of the tested tissues. Expression of *SfruGR1* and *SfruGR2* was significantly higher in male antennae than in female antennae or in larval a-m, and expression of *SfruGR3* and *SfruGR9* was tended to be higher in female antennae than in male antennae or in larval a-m. *SfruGR3* was also highly expressed in the larval a-m, i.e., its expression was slightly lower (but not significantly lower) in the larval a-m than in the female antennae (Figure 4).

### Candidate IRs/iGluRs

**FIGURE 5:**
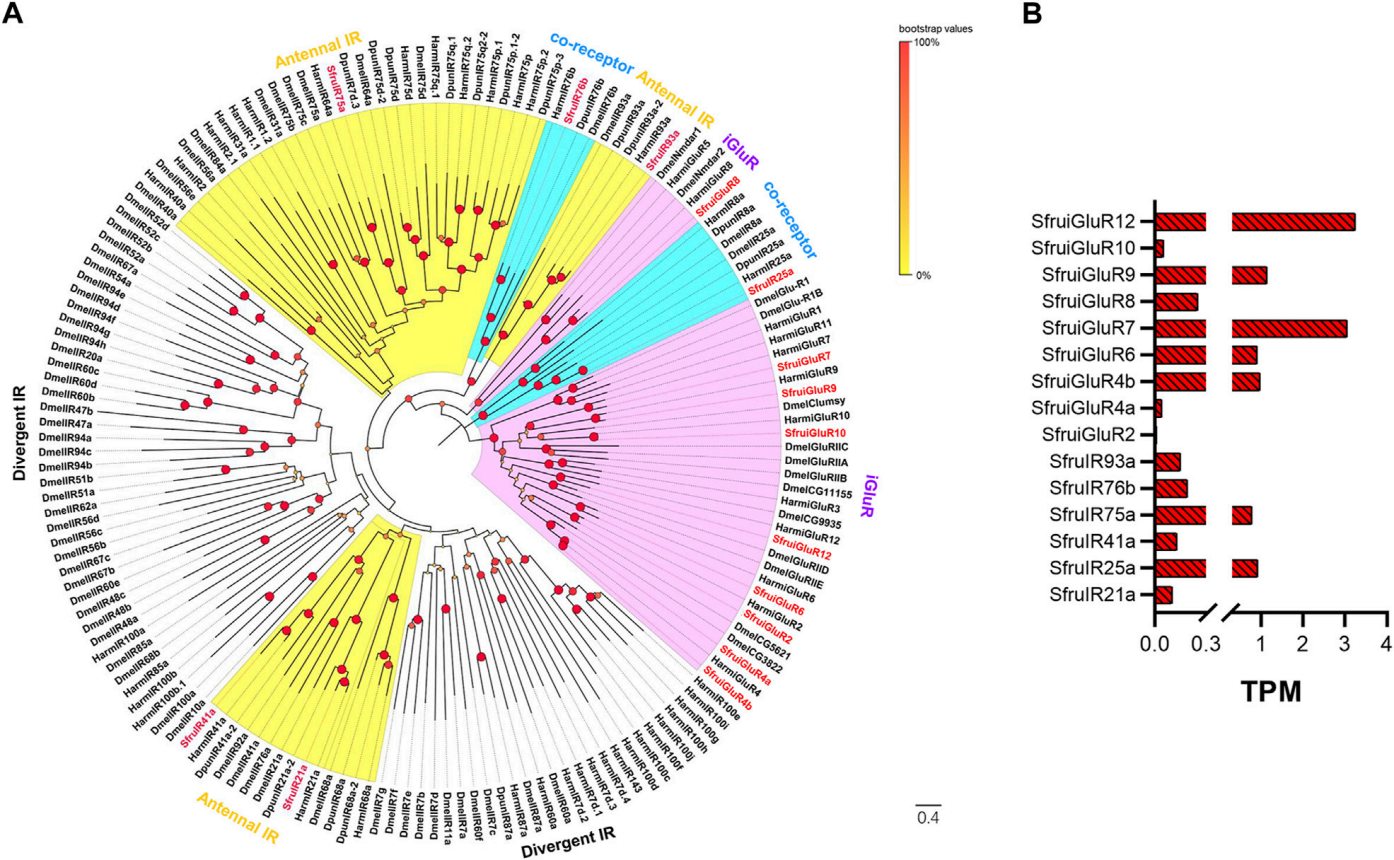
Candidate IRs/iGluRs of *S*. *frugiperda* larvae **(A)** Phylogenetic relationships of IRs/iGluRs from *S. frugiperda* (Sfru, this study), *H*. *armigera* (Harm), *D. melanogaster* (Dmel), and *D. punctatus* (Dpun). The neighbor-joining tree was constructed using MEGA11 (1000 bootstrap replicates). The tree was rooted with the conservative iGluRs genes. The IR co-receptor branches are highlighted in blue; the ionotropic glutamate receptor (iGluRs) branches are highlighted in purple; the “antennal IR” branches are highlighted in yellow; the “divergent IR” branches are not highlighted. **(B)** TPM values of candidate IRs/iGluRs in antennae and maxillae of *S*. *frugiperda* larvae.

According to the transcriptome analysis, a total of 6 putative SfruIRs and 9 putative SfruiGluRs were identified (Table 1). Five of the 6 putative SfruIRs had full-length ORFs, and 5 of the 15 SfruIRs/iGluRs lacked a C-terminal (SfruiGluR2/7), an N-terminal (SfruIR93a/SfruiGluR8), or both a C- and an N-terminal (SfruiGluR12) (Supplementary Table S5). A phylogenetic tree built with these SfruIRs/iGluRs and homologs from *D*. *melanogaster*, *H*. *armigera*, and *D. punctatus* showed that the candidate SfruIR25a and SfruIR76b were distributed in the co-receptor IR25a and IR76b lineages, respectively. Four IRs (SfruIR21a/41a/75a/93a) were clustered in the “antennal IRs” sub-group. None of the SfruIRs appeared in the “divergent IRs” subgroup (Figure 5A).

TPM calculation demonstrated that the most enriched SfruIR/iGluR in the *S*. *frugiperda* larval a-m was *SfruiGluR12* (TPM = 3.05), followed by *SfruiGluR7* (TPM = 2.95) and *SfruiGluR9* (TPM = 1.13). The co-receptor *SfruIR25a* had a low TPM value of 0.91, which was higher than that of the co-receptor *SfruIR76b* (TPM = 0.19) (Table1; Figure 5B).

**FIGURE 6:**
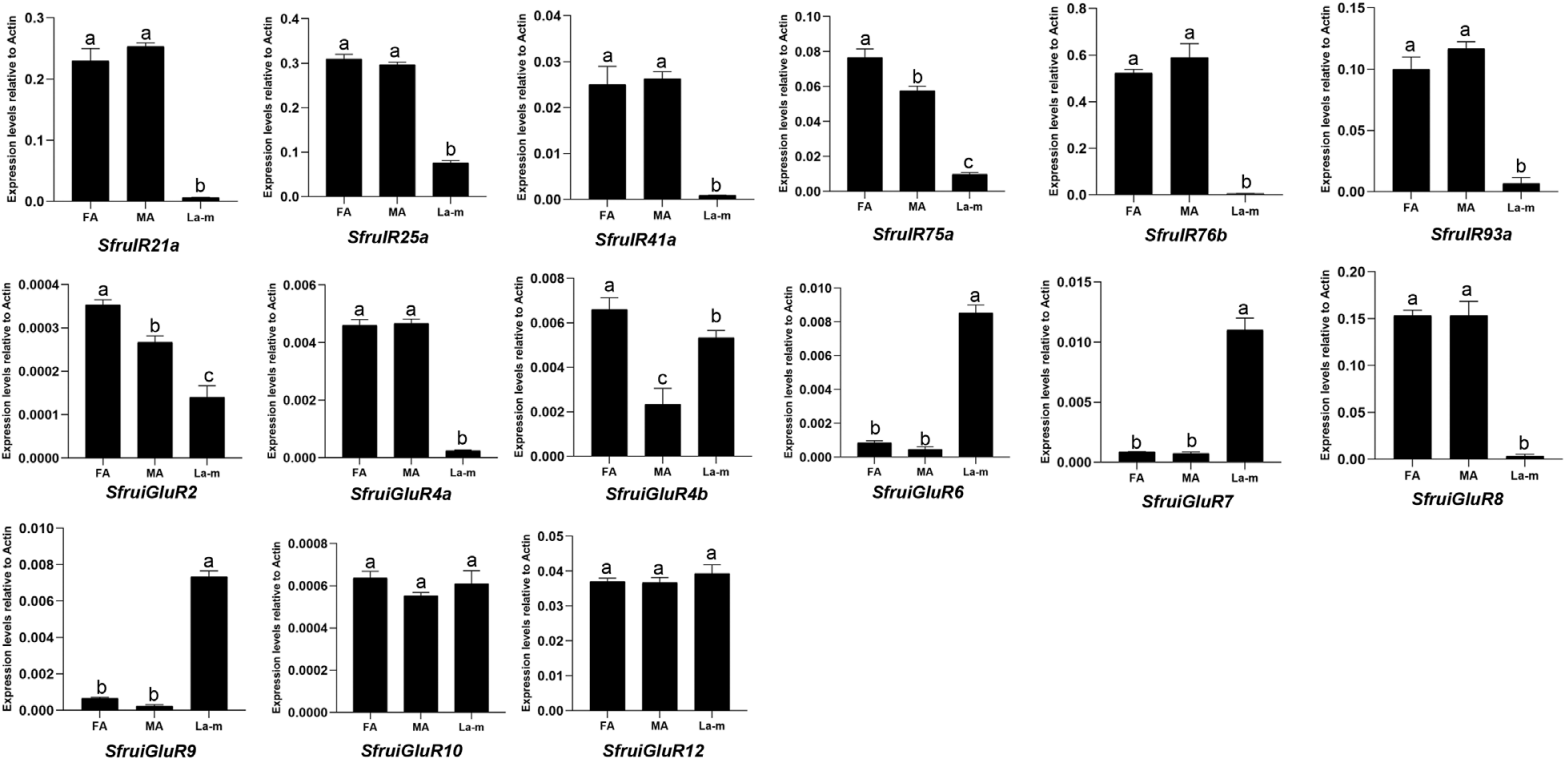
Expression profiles (as determined by RT-qPCR analysis) of candidate *SfruIRs*/*iGluRs* genes in *S. frugiperda* male antennae (MA), female antennae (FA), and larval antennae and maxillae (L a-m). Values are means ± SE. Different letters indicate significant differences (Tukey’s multiple comparison test.after a one-way ANOVA, *p* < 0.05, *n* = 3).

According to RT-qPCR analysis, the expression of the 6 candidate *SfruIR* genes was greater in the antennae of *S*. *frugiperda* adult females and males than in the larval a-m. The expression of *SfruiGluR2*/*4a*/*4b*/*8* was also greater in the antennae of *S*. *frugiperda* adult females and males than in the larval a-m. Although expression levels of SfruiGluR*10*/*12* were similar between adult antennae and the larval a-m, the expression levels of *SfruiGluR6*/*7*/*9* were significantly higher in the larval a-m than in the antennae of adult females and males (Figure 6).

### Expression profiles of chemosensory receptors in different stages of *S*. *frugiperda* larvae

**FIGURE 7:**
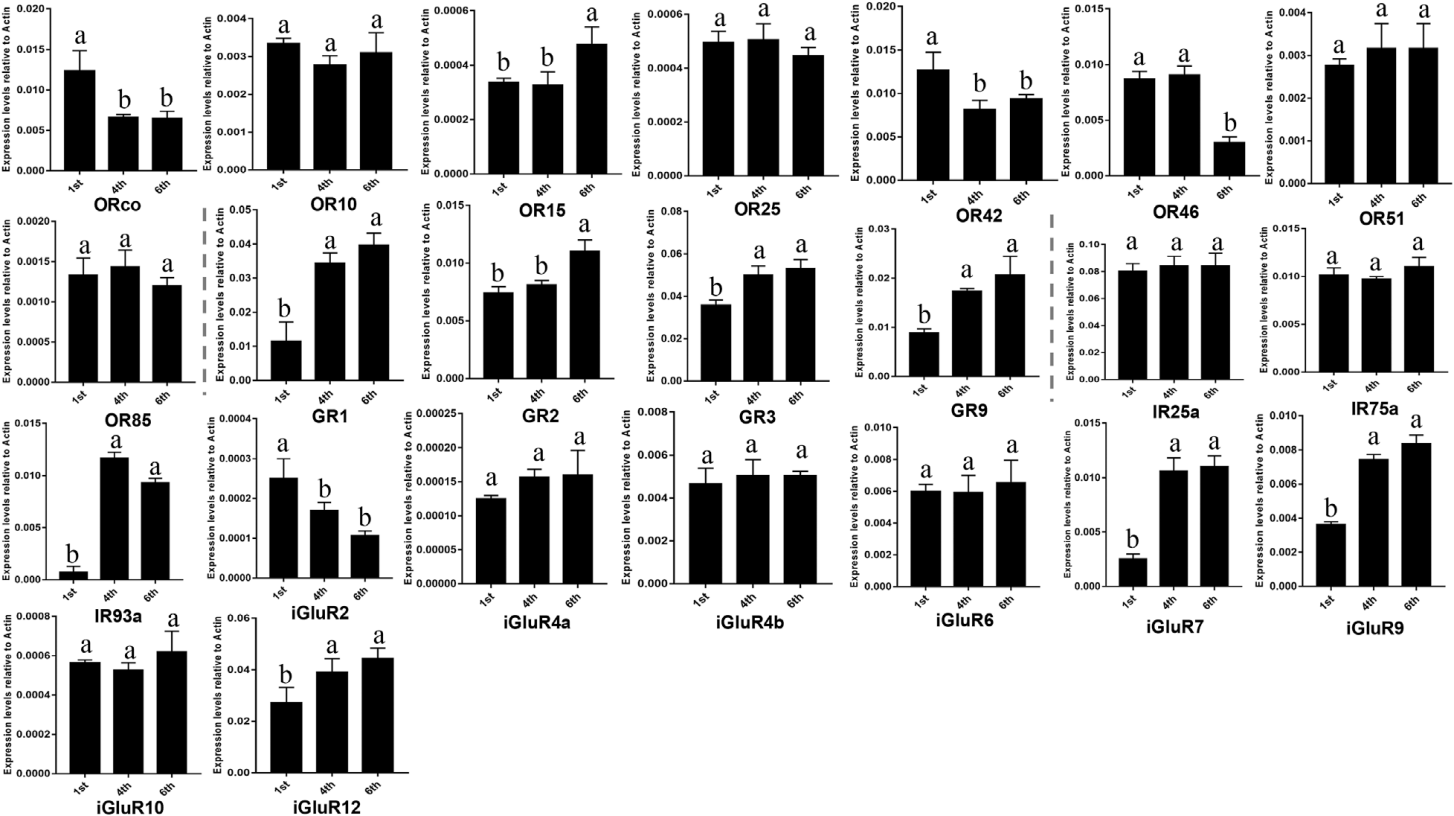
Expression profiles of chemosensory receptors in different stages of *S*. *frugiperda* larvae. RT-qPCR analyses of the genes displaying larval a-m expression were conducted in the first (1st) instar larval head, the fourth (4th) instar larval antennae and maxillae, and the sixth (6th) instar larval antennae and maxillae. Values are means ± SE. Different letters indicate significant differences (Tukey’s multiple comparison test after a one-way ANOVA, *p* < 0.05, *n* = 3).

To reveal expression levels of the chemosensory receptors during *S*. *frugiperda* larval development, we selected three stages, first, fourth, and sixth instars, to cover the larval developmental period. In total, 23 chemosensory receptor genes including 8 *SfruORs*, 4 *SfruGRs*, and 11 *SfruIRs*/*iGluRs* showed larval a-m expression were measured using RT-qPCR (not including the genes of which the expression in larval a-m was barely detected). According to the RT-qPCR results, 2 *SfruORs* (*SfruORco*/*SfruOR42*) and 1 *SfruiGluR* (*SfruiGluR2*) displayed higher expression in the first instar than in other two instars. While 3 *SfruGRs* (*SfruGR1*/*3*/*9*) and 4 *SfruIR*/*iGluRs* (*SfruIR93a* and *SfruiGluR7*/*9*/*12*) displayed higher expression in the fourth and sixth instars than in the first instar. In comparison, the expression of *SfruOR15* and *SfruGR2* was higher in the sixth instar than in other two instars, and the expression of *SfruOR46* was higher in the first and fourth instars than in the sixth instar. Other measured genes exhibited similar expression levels among the three stages (Figure 7).

## Discussion

Despite the wide distribution of *S*. *frugiperda* and the severe damage to crops caused by its larvae, information concerning the larval chemosensory system is limited. In the current study, we focused on three groups of receptors (ORs, GRs, and IRs) because of their potential importance to the chemosensory system of *S*. *frugiperda* larvae and therefore to the development of new pest control strategies.

In this study, we analyzed the transcriptome of the *S*. *frugiperda* larval a-m by *de novo* sequencing. The total number of SfruORs identified here (11) is close to the number (16) of ORs found in the larval transcriptome of *H*. *armigera* (Di et al., 2017) but is much lower than the number of putative ORs reported in the larval transcriptome of *S. littoralis* (22) and *S*. *litura* (22) (Poivet et al., 2013; Li et al., 2021). The large difference in the numbers of ORs found in our study of *S*. *frugiperda* than in previous studies of *S. littoralis* and *S*. *litura* may have resulted from the sample collection methods. In the current study of *S*. *frugiperda*, the larval a-m were collected on the 2nd day in the 6th instar; in contrast, the larval a-m were collected in the 4th instar of *S*. *littoralis* (Poivet et al., 2013), and entire heads were collected from 1- to 6-day-old larvae of *S*. *litura* (Li et al., 2021). Furthermore, the assembled larval head transcriptome of *S*. *littoralis* revealed 34 ORs in the 1st instar but only 18 ORs in the 4th instar (Revadi et al., 2021).

Although we found 11 ORs in *S*. *frugiperda* larvae, 26 ORs were recently detected in the transcriptome of antennae of *S*. *frugiperda* adults (Qiu et al., 2020). This difference between the number of ORs in *S*. *frugiperda* larvae and adults is consistent with that observed for ORs in other lepidopteran species. For example, *B*. *mori* expressed 23 ORs in larvae but 35 in adults (Tanaka et al., 2009); *H*. *armigera* expressed 16 ORs in larvae but 47 in adults (Liu et al., 2012; Di et al., 2017); *S*. *littoralis* expressed 22 ORs in larvae but 47 in adults (Poivet et al., 2013); and *S*. *litura* expressed 22 ORs in larvae but 60 in adults (Li et al., 2021). These differences may be due to differences in the physiological and behavioral characteristics of larvae vs. adults regardless of species.

Consistent with the expression profile of *ORco* genes reported for other lepidopteran species (Di et al., 2017; de Fouchier et al., 2018), we found that the *SfruORco* was highly expressed in both the adult antennae and the larval a-m. This indicated the ability of olfaction in both the larvae and adults of *S*. *frugiperda*. In the study of model insect *B*. *mori*, deletion of the *ORco* gene using CRISPR/Cas9 system demonstrated that the homozygous mutant of adults was unable to respond to bombykol or bombykal, and larval feeding behavior assays revealed that the mutant larvae displayed defective selection for mulberry leaves and the mulberry leave volatile *cis*-jasmone (Liu et al., 2017). Like the OR expression profiles reported in other lepidopteran species (Di et al., 2017; de Fouchier et al., 2018; Li et al., 2021), expression of most of the annotated *SfruOR*s in our study of *S*. *frugiperda* was greater in adult antennae (male/female) than in the larval a-m. The *SfruOR*s (*SfruOR11*/*12*/*20*) that displayed adult antennae-specific expression profiles may be involved in the olfaction of *S*. *frugiperda* adults. For example, HarmOR27, an ortholog of SfruOR20, mainly expressed in the adult antennae of male and female *H*. *armigera*, was found triggering butyl salicylate when heterologously expressed in *Drosophila* empty neurons (Guo et al., 2021). The *SfruOR*s (*SfruOR10* and *SfruOR51*) that showed similar transcript levels in the larval a-m and adult antennae may have olfactory functions in both developmental stages. As an ortholog of *SfruOR51*, *SlitOR31* showed expression in the antennae of both adult and larval *S*. *littoralis* (De Fouchier et al., 2018). Heterologous expression of this receptors in *Drosophila* exhibited narrow tuning to the plant volatile eugenol that commonly emitted by flowers (De Fouchier et al., 2017).

SfruOR42, an ortholog of HarmOR42 (amino acid identity: 86%), was identified in the current study. A previous study found that HarmOR42 was expressed in the antennae of *H*. *armigera* larvae and that this receptor responded strongly to the larval attractant phenylacetaldehyde in the *Xenopus* oocyte recording system (Di et al., 2017). The exact function of SfruOR42 (whether or not *S*. *frugiperda* larvae use it to detect phenylacetaldehyde) remains to be determined.

Because insect larvae do not mate, detection of sex pheromones by insect larvae has seldom been reported. However, research has shown that *S. littoralis* larvae were more attracted to a food source when it contained species-specific sex pheromone components (Poivet et al., 2012). Similar results were reported for larvae of *Plutella xylostella* (Zhu et al., 2016) and *S*. *litura* (Han et al., 2022). In the current study, we identified a putative pheromone receptor *SfruOR11*, which was clustered in the lepidopteran pheromone receptor OR11 clade. RT-qPCR analysis showed that *SfruOR11* was expressed in both adult antennae and the larval a-m, with the expression level significantly higher in male antennae than in female antennae or the larval a-m. Given that the specific ligands for the OR11 clade in lepidopterans are still unknown (Yang and Wang, 2021), the role of sex pheromone perception by *S*. *frugiperda* larvae remains to be elucidated.

We identified three GRs (SfruGR1/2/3) that are homologous with *B*. *mori* or *H*. *armigera* CO_2_ receptors. It is well known that adult moths can use CO_2_ concentration to evaluate flower profitability (Thom et al., 2004). Specialized neurons that sense CO_2_ are predominantly enriched in the labial palps in adult moths, and three GRs, GR1/2/3, are responsible for the detection of CO_2_ (Xu and Anderson, 2015; Ning et al., 2016). In this study, we found that three CO_2_ receptor genes were abundantly expressed in the male and female adult antennae, and that *SfurGR3* was also highly expressed in the larval a-m. Similar expression characteristics have been reported in *H*. *armigera* (Xu and Anderson, 2015). Because little is known about the ability of lepidopteran larvae to detect CO_2_, functional investigation is needed of SfruGR1/2/3 in the larval a-m of *S*. *frugiperda*. Another GR, SfruGR9, the homolog of *H*. *armigera* GR9 (HarmGR9) and *B*. *mori* GR9 (BmorGR9), was detected in the current study. HarmGR9 was previously found to have high transcription levels in *H*. *armigera* female antennae and larval foreguts, and to respond specifically to *D*-fructose (Jiang et al., 2015). Similarly, BmorGR9 in *B*. *mori* was identified as a fructose receptor (Sato et al., 2011). In the current study, *SfruGR9* was expressed in both adults and larvae (although the expression level was higher in female antennae than in the other tissues), which is reasonable because both *S*. *frugiperda* larvae and adults can detect fructose. Further research is needed to determine the roles of SfruGR9 in adult antennae (especially female antennae) and in the larval chemosensory organs of *S*. *frugiperda*.

Insect IRs are expressed in chemosensory organs and other tissues involved in olfaction, gustation, and in the sensing of humidity and temperature (Rytz et al., 2013; Robertson, 2019; Hou et al., 2022). In the current study, four “antennal IRs” (SfruIR21a/41a/75a/93a) and two IR co-receptors (SfruIR25a/76b) were annotated in the larval a-m of *S*. *frugiperda*. RT-qPCR demonstrated that all of these annotated “antennal IRs” and co-receptors were highly expressed in adult antennae of both sexes; these results are consistent with those reported for *H*. *armigera* and *Agrotis segetum* (Liu et al., 2014; Hou et al., 2022). *SfruIR25a* and *SfruIR75a* were also highly expressed in that larval a-m (although at a significantly lower level than in adult antennae), indicating that these IRs are involved in the chemosensory reception in both larvae and adults of *S*. *frugiperda*. In *D*. *melanogaster*, IR25a and IR93a have been reported to be indispensable for the detection of cool temperatures by IR21a (Ni et al., 2016). The functional properties of their orthologs (SfruIR21a/25a/93a) identified in the current study remain to be investigated.

Ionotropic glutamate receptors (iGluRs) are localized on the surface of neuron synapses and are involved in the transmission of signals in nervous systems (Benton et al., 2006; Abuin et al., 2011). Three subfamilies of iGluRs, including NMDA (N-methyl-D-aspartate), kainate, and AMPA (α-amino-3- hydroxy-5-methyl-4-isoxazolepropionic acid) have been well studied in their structural features and biophysical functions (Mayer, 2011; Rytz et al., 2013). In the current study, we identified 9 iGluRs in the larval a-m of *S*. *frugiperda*. We found that some *iGluRs* (*SfruiGluR2*/*4a*/*4b*/*8*) were mainly expressed in adult antennae, that expression of *SfruiGluR2*/*4b* was higher in female than in male antennae, that expression of *SfruiGluR6*/*7*/*9* was higher in the larval a-m than in adult antennae, and that expression of *SfruiGluR10*/*12* was similar in adult antennae and the larval a-m. These differences in expression levels of *iGluRs* are probably related to their importance in different tissues/stages. Among all of the annotated *SfruIRs*/*iGluRs*, the expression level was highest for *SfruiGluR7*. RT-qPCR analyses also showed that *SfruiGluR7* was much more enriched in the larval a-m than in adult antennae. We therefore suggest that this gene may be especially important in the transmission of neuron signals in *S*. *frugiperda* larvae which should be further experimentally validated.

Expression levels of 23 larval a-m expressed chemosensory receptor genes during larval development were further analyzed using RT-qPCR. Three larval stages (first, fourth, and sixth instars) were selected to cover the larval developmental period (early, middle, and gluttonous stage) (Dai et al., 2020; Wang et al., 2020; Xie et al., 2021). We found that the expression of 13 chemosensory receptor genes including 4 *SfruORs*, 4 *SfruGRs*, and 5 *SfruIRs*/*iGluRs* varied significantly among the three larval stages. Similar findings had been reported by other researchers. Measurements of the dynamic expression of *S*. *litura* chemosensory genes demonstrated that some of the genes showed differential expression during larval developmental stages (Li et al., 2021). Studies on the expression profiles of ORs in the larvae of *S*. *littoralis* between the first and fourth instar revealed that some of the ORs exhibited instar-biased expression (Revadi et al., 2021). Differential expression investigation of chemosensory receptor genes during larval development of *S*. *frugiperda* in this study provides a good basis to understand odor-guided behavioral traits of the larvae, and to analyze the function of chemosensory receptor genes during larval feeding (Yactayo-Chang et al., 2021)*.*

Furthermore, we found that expression levels of 4 *SfruGR* genes (*SfruGR1*/*2*/*3*/*9*) were higher in the sixth instar than in the first and fourth instars. While the expression of *SfruORco* was higher in the first instar than in the fourth and sixth instars. Xu and Anderson (2015) reported that expression levels of CO_2_ GR genes (*HarmGR1*/*2*/*3*) from the cotton bollworm *H*. *armigera* were higher in the fifth instar than in the third instar. Transcriptomic analysis combined with RT-PCR demonstrated that the *ORco* gene of *S*. *littoralis* had higher expression in the heads of first instar larvae than in the fourth instar (Revadi et al., 2021). Correlations between the expression levels of these genes and the larval development stages, however, need to be confirmed in more moth species. And functional importance of such expression patterns during larval feeding is worth further investigation.

In conclusion, this work represents the first annotation and expression profile analysis of the chemosensory receptors in larval antennae and maxillae of the lepidopteran pest *S*. *frugiperda*. Our findings provide a foundation for future studies of the functions of the chemosensory receptors in *S*. *frugiperda* larvae.

## Data Availability

The datasets presented in this study can be found in online repositories. The names of the repository/repositories and accession number(s) can be found in the article/Supplementary Material.
