# Supplementary material for: Candidate chemosensory receptors in the antennae and maxillae of Spodoptera frugiperda (J. E. Smith) larvae
Source: Front Physiol. 2022 Sep 15;13:970915. doi: 10.3389/fphys.2022.970915 (PMC9520170; doi:10.3389/fphys.2022.970915)
Supplement: Supplementary file 1 [file DataSheet1.zip › Supplementary Files/Supplementary figure legends.docx]

**Supplementary figure legends**

**Figure S1.** Homologous species distribution of *S. frugiperda* unigenes in non-redundancy protein database.

**Figure S2**. Functional annotation of *S. frugiperda* larval antennae and maxillae based on the gene ontology (GO) categorization.
