# Supplementary material for: Candidate chemosensory receptors in the antennae and maxillae of Spodoptera frugiperda (J. E. Smith) larvae
Source: Front Physiol. 2022 Sep 15;13:970915. doi: 10.3389/fphys.2022.970915 (PMC9520170; doi:10.3389/fphys.2022.970915)
Supplement: Supplementary file 1 [file DataSheet1.zip › Supplementary Files/Table S2.docx]

**Table S2**. Primers for real-time quantitative-PCR of candidate *OR*s, *GRs*, and *IRs/iGluRs*.

| **ID** | **Forward primer (5'to 3')** | **Reverse primer (5' to 3')** |
| --- | --- | --- |
| ***ORs*** |  |  |
| *SfruORco* | TAATGGACTGACTCCGAAAC | TGGTAAGCCAGCAAAGTG |
| *SfruOR10* | GATAAGGATTGGGAAGTCA | CAACAGAAGCCACAGAAA |
| *SfruOR11* | GGAAAGAAGCGGGAGGAT | GCCGTCATTTGCGAACAC |
| *SfruOR12* | TGTTCTCCGACATCATTCC | GTACGCCACCTCGTTCCA |
| *SfruOR15* | TTGGTACGCCGATGATAT | TACGCTGTTGTAAGGATTGA |
| *SfruOR20* | CCAATCGGATGATGAAGA | CCAGGCAGTATGTGAGGA |
| *SfruOR25* | CGAGAAGACGGGACAAGG | GTGAGAAATGCGGCGAAC |
| *SfruOR42* | AGACTGTCGGCTTGTTGT | CACCTTCTCGGATATTGT |
| *SfruOR46* | ACCGTTGCCATTATCTGC | ACTTGGTGTCCTGCTCGT |
| *SfruOR51* | ATATTCGGTGAAGCAATG | GTAAATAAACAACTGGGCTA |
| *SfruOR85* | TGATAATGAGCACTACGACGAG | TAGGAGCACAATAACGAACACT |
| ***GRs*** |  |  |
| *SfruGR1* | GGTAATCTTCTTCACGACTAC | TCCGAGGTTCACATTCAG |
| *SfruGR2* | GTATCATTATCCGCTCATATCC | ATCCACGATCAGTTCTACAG |
| *SfruGR3* | GGAGTGCTGCCCATTACG | GAACTTGCCTTCCGCTGT |
| *SfruGR9* | AGGCGGACAATACTCTTTA | AGTACGACGAACCACAGC |
| ***IRs*** |  |  |
| *SfruIR21a* | AGGTTACGCTGGTCACAG | ATTAGGCTCACGAGGTTC |
| *SfruIR25a* | ATAAGGGACGAGCAAGCA | TCAGCCATGACAGACAGC |
| *SfruIR41a* | ACCGTATGTGCTGTTAGAC | TCTTCTACAACGCTTCCAAT |
| *SfruIR75a* | GCCCAGGATAGTGAACCA | TCACCATGCCATCGAAAC |
| *SfruIR76b* | TGCGGAACAGTGATCTTA | AAACAGCGAGTCGTATGG |
| *SfruIR93a* | ATGGATTCAGAGGGAAGG | GCATCATATCAGTGGTCGT |
| *SfruiGluR2* | TAGAAGAGGGTTCCAATG | ATGTTCCCACCTTATCCA |
| *SfruiGluR4a* | GACGGCATGATAAGGGAATT | CGGCGATAGGAACGAGAA |
| *SfruiGluR4b* | GACGGCATGATAAGGGAATT | CGGCGATAGGAACGAGAA |
| *SfruiGluR6* | GGACTGCTGGACTCTAAG | CTGTTTCTGTTCCTCGTC |
| *SfruiGluR7* | TGGCTGAAGTGTCTGTGA | TAGAGGCGTCCATTGATA |
| *SfruiGluR8* | TCGTTTGGGCTGTTCTATT | AGTGAGTGGCCTCGCTAT |
| *SfruiGluR9* | GCATCTGCGACACTACTT | TACGAAACTGCTATCATCC |
| *SfruiGluR10* | AATGGGAAGTCGGTGAAG | AGCGGTCTGATGTTTCGT |
| *SfruiGluR12* | GGGACTACGGGTCCAAAGACAA | CAGGATGCTGATGCCGAGGTT |
| *SfruActin* | TACTCCTAAGCCTGTTGATG | TTATGTCATGGTGCCGAAT |
