# Supplementary material for: Candidate chemosensory receptors in the antennae and maxillae of Spodoptera frugiperda (J. E. Smith) larvae
Source: Front Physiol. 2022 Sep 15;13:970915. doi: 10.3389/fphys.2022.970915 (PMC9520170; doi:10.3389/fphys.2022.970915)
Supplement: Supplementary file 1 [file DataSheet1.zip › Supplementary Files/Table S3.docx]

Table S3: Evaluation of sequencing data of larval *S. frugiperda* samples.

| Repeat | Read Number | Base Number | GC Content | ≥Q30 (%) |
| --- | --- | --- | --- | --- |
| 1 | 56,634,546 | 8,495,181,900 | 45.93% | 89.87% |
| 2 | 52,905,990 | 7,935,898,500 | 47.20% | 91.19% |
| 3 | 60,048,292 | 9,007,243,800 | 47.83% | 89.82% |
|  | | | | |
