# Supplementary material for: Candidate chemosensory receptors in the antennae and maxillae of Spodoptera frugiperda (J. E. Smith) larvae
Source: Front Physiol. 2022 Sep 15;13:970915. doi: 10.3389/fphys.2022.970915 (PMC9520170; doi:10.3389/fphys.2022.970915)
Supplement: Supplementary file 1 [file DataSheet1.zip › Supplementary Files/Table S4 .docx]

Table S4. Assembly summary of transcriptome in larval antennae and maxillae of *S*. *frugiperda*.

| Length Range | Transcript | Unigene |
| --- | --- | --- |
| ≥500bp | 107,221 (44.48%) | 30,912 (32.13%) |
| ≥1000bp | 51,849 (21.51%) | 14,171(14.73%) |
| Total Number | 241,014 | 96,197 |
| Total Length | 182,243,685 | 60,887,852 |
| N50 Length | 1171 | 972 |
| Mean Length | 756 | 633 |
