# Supplementary figures and images for: Candidate chemosensory receptors in the antennae and maxillae of Spodoptera frugiperda (J. E. Smith) larvae
Source: Front Physiol. 2022 Sep 15;13:970915. doi: 10.3389/fphys.2022.970915 (PMC9520170; doi:10.3389/fphys.2022.970915)

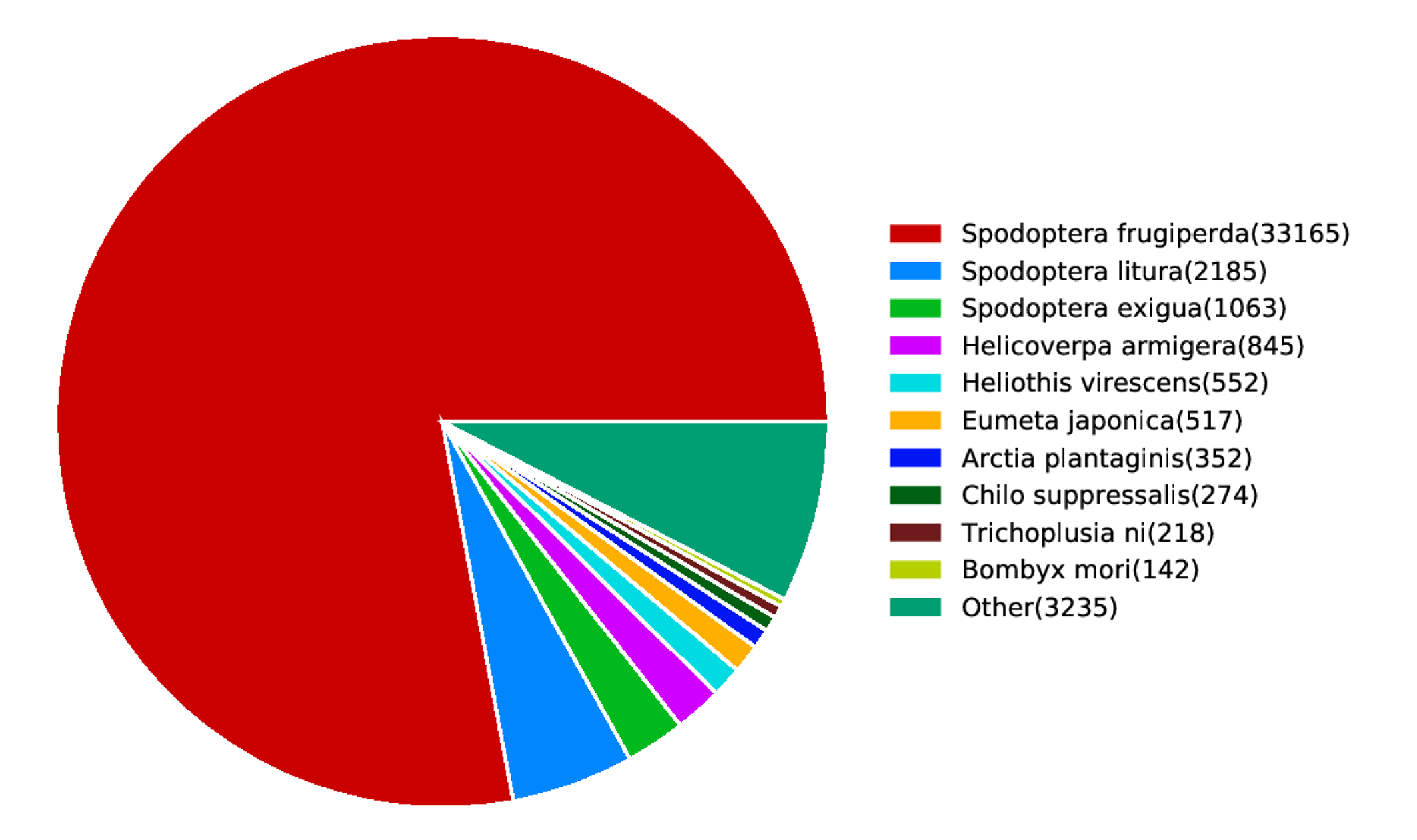

Supplement: Supplementary file 1 [file DataSheet1.zip › Supplementary Files/Figure S1.tif]

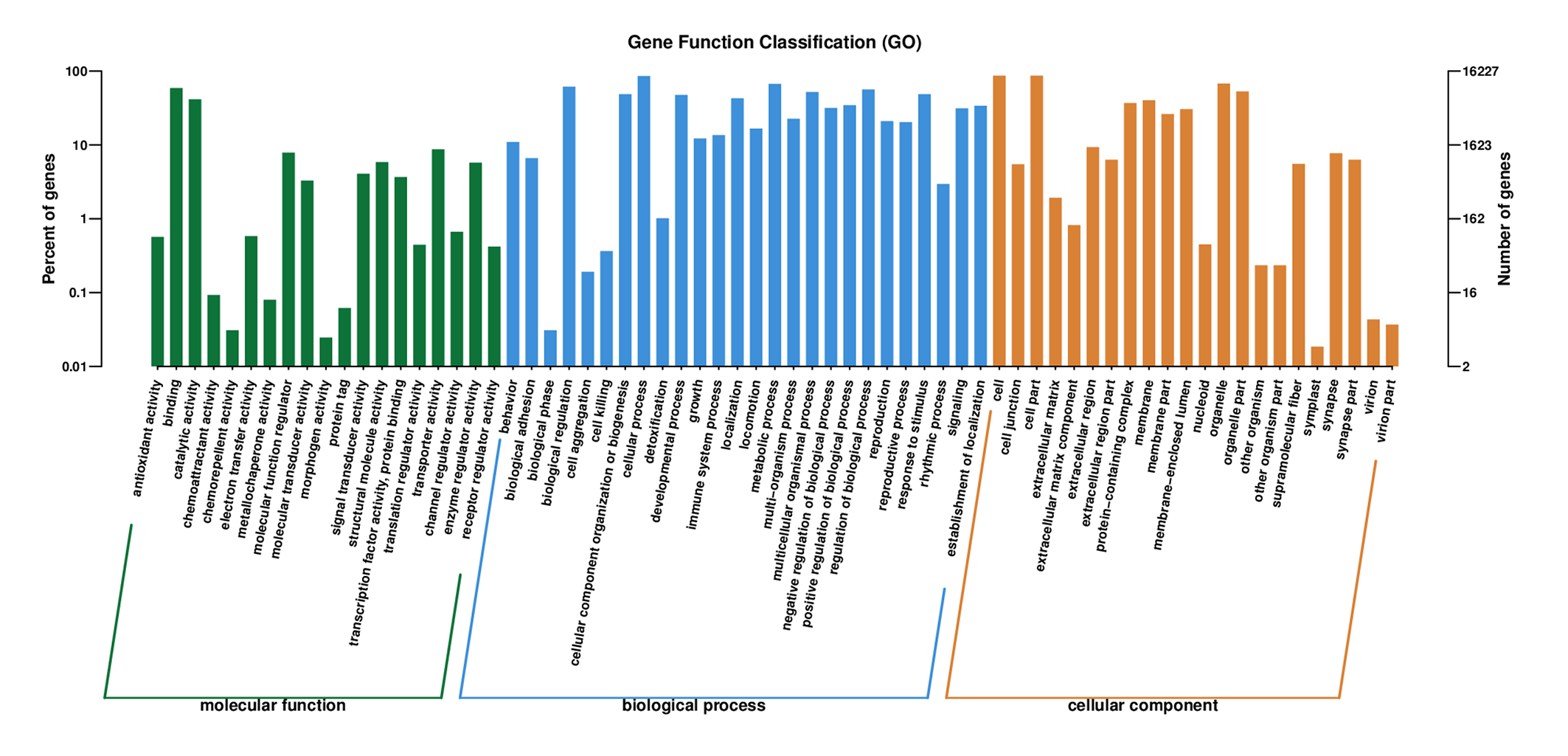

Supplement: Supplementary file 1 [file DataSheet1.zip › Supplementary Files/Figure S2.tif]
